# Age and school-segment difference in daily sedentary behavior and physical activity among student (9–23 years): a cross-sectional accelerometer-based survey

**DOI:** 10.3389/fped.2023.1202427

**Published:** 2023-07-17

**Authors:** Yijuan Lu, Kehong Yu, Mengjie Zhai, Pan Ma

**Affiliations:** ^1^ School of Physical Education, Hangzhou Normal University, Hangzhou, China; ^2^ Department of Sport and Exercise Sciences, College of Education, Zhejiang University, Hangzhou, China; ^3^ A Center for Sports Modernization and Development, Zhejiang University, Hangzhou, China; ^4^ Liangzhu Second Middle School, Hangzhou, China; ^5^ Linping First Middle School, Hangzhou, China

**Keywords:** 24-hour behavior, adolescent, China, children, actigraph

## Abstract

This study is cross-sectional in nature and aims to investigate and track sedentary behavior (SB) and physical activity among student (aged 9–23 years) for seven consecutive days using an accelerometer. It also intends to analyze the current status of the daily activities of students using age and school-segment differences. The study recruits a total of 384 students [age: 14.41 ± 3.52 years; body mass index (BMI): 19.66 ± 3.67] from four schools out of which 180 (46.88%) were male. The study uses the means and standard deviations for statistical analysis and independent sample *t*-tests to determine gender differences. Analysis of covariance is used to determine whether or not daily SB and physical activity were statistically significant students according to gender and school segment followed by LSD *post hoc* tests for multiple comparisons. The results demonstrate that students were less physically active [moderate- to vigorous-intensity physical activity (MVPA):60.4 ± 23.48 min/day] and more sedentary (598.47 ± 162.63 min/day). The sedentary time of the students displays an inverted U-trend, and their participation in MVPA exhibits a W-shape. After controlling for BMI, the results of ANCOVA point to a significant school-segment effect (*p* < 0.001) for SB (*F* = 83, *η_p_*^2^ = 0.4) and physical activity (low-intensity physical activity: *F* = 108.61, *η_p_*^2^ = 0.47; MPA: *F* = 401.65, *η_p_*^2^ = 0.76; high-intensity physical activity: *F* = 88.43, *η_p_*^2^ = 0.42; MVPA: *F* = 118.42, *η_p_*^2^ = 0.49). Based on the behavioral characteristics of students across school segments, this study concluded that interventions targeting students' physical activity and physical health should be school segment specific. The results of the study provide a basis for future analysis of factors influencing students' physical activity behaviors across school segments and for proposing targeted intervention strategies for the future.

## 1. Introduction

Changes in modern lifestyle have led to a pronounced decrease in human physical activity (hereafter, physical activity). Numerous studies have demonstrated that the decreased amount of physical activity among students is the most important reason for the decline in physical fitness. Moreover, physical inactivity among students has become a global health problem. To promote health, the World Health Organization (WHO) has recommended that children and adolescents (aged 5–17 years) engage in 60 min of moderate- to vigorous-intensity physical activity (MVPA) (1). Studies on international instrumental measurements of MVPA in children have illustrated that no more than 45% of students meet the daily recommendations of the WHO (2, 3), where only 8% of adolescents meet the standard (1). In Finland, 90% of girls and 77% of boys have not met the daily recommended amount (MVPA ≥ 60 min/day) in a self-report (4). In several European countries, accelerometer measurements of children aged 10–12 years indicated that only 4.6% of girls as well as 16.8% of boys met the recommended amount (5).

In China, the current state of physical activity among students is also worrisome. According to the data of the 2010 National Physical Fitness and Health Test on the physical activity levels (PALs) of 166,812 school children aged 9–18 years in 30 provinces in China, only 22.7% of the students reported having achieved the recommended 60 min of physical activity per day (6). Moreover, data by age group demonstrated that 32.7%, 20.7%, and 12.5% of students aged 9–13, 13–16, and 16–19 years, respectively, achieved the recommended amount of physical activity. The data have reflected a trend of decreasing PALs among students with the increase in age. Wang (7) used an accelerometer to test the daily physical activity of 2,897 children and adolescents in 11 cities in China and determined their levels of physical activity from ages 9 to 17 years. Specifically, only 5.6% of the students met the WHO recommendation (MVPA ≥ 60 min/day), and, in particular, only 1.9% of the group of female students met the standard requirement. In addition to the insufficient time for engaging in MVPA, the Chinese students displayed increased sedentary hours. Dearth-Wesley et al. (8) conducted a longitudinal study and demonstrated that the proportion of students with more than 2 h of daily sedentary time during leisure time reached 58.9%.

Inadequate physical activity is strongly associated with health in students. Wu et al. (9) found that high levels of physical activity were associated with better health-related quality of life after a systematic review of 31 papers. Moreover, increased time spent on SB was associated with low quality of life in children and adolescents. The dose–response relationship among physical activity, SB, and health-related quality of life suggested that high frequencies of physical activity or less time spent on SB is associated with better health-related quality of life. For this reason, China proposed an MVPA of no less than 60 min/day for students to promote their health (10). Scholars demonstrated that regular physical activity provided many health benefits for children and adolescents such as weight loss (11). In addition, health benefits gained during childhood and adolescence can persist into later life; for example, children with high levels of physical activity early in life exhibit high levels of bone mass density in later childhood (12). A 21-year follow-up survey illustrated that groups of children and adolescents with long-term physical activity obtained low risks of developing metabolic syndrome in adulthood (13). In addition, the development of exercise habits in children and adolescents is particularly important. Previous studies indicated that children and adolescents with low levels of physical activity also have low levels of physical activity in adulthood (14, 15). Thus, the urgent need emerged to effectively curb the prevalence of physical inactivity among students and to improve levels of physical activity. Alternatively, physical inactivity limits the improvement of the physical health of students, and an increasing number of evidence-based reports due to the increased research on physical activity supported the health-promoting function of physical activity (16).

Given the health benefits of regular physical activity, we might wonder why students are not active at recommended levels. There are many common barriers that affect students' physical activity. There are many common barriers to physical activity, such as lack of time, lack of social support, lack of energy, lack of motivation, fear of injury, lack of skill, lack of facilities, and weather conditions (17). Understanding common barriers to physical activity and developing strategies to overcome them may help students make physical activity a part of their daily lives.

Physical activity among students is not a simple isolated behavior; a combination of physiological, psychological, and social factors determines the behavior of participating and sustaining physical activity, and the complexity of such a behavior determines the diversity and relevance of intervention strategies. The social–ecological model proposed by Bronfenbrenner in 1977 (18) is a widely used and comprehensive theoretical framework for analyzing the factors that influence physical activity and guide the design of intervention strategies and programs. The model emphasizes that a combination of individual and environmental factors, including those at the individual (attitudes, cognition, and self-efficacy), interpersonal (social support and role models), organizational (factors related to the major places of daily activity, e.g., schools and families), community (physical environment and staffing), and policy (national or local health promotion policies and resources) levels. Thus, the factors that influence physical activity are multi-dimensional.

In this regard, the multidimensional and interacting changes in the various influencing factors of the physical activity of students determine the complexity of its promotion. Notably, in the social–ecological model, the individual factor lies at the core level, and other levels vary according to the characteristics of the individual factor. Students are a collective term that generalizes childhood and adolescence, and the variation in key factors that influence physical activity within this group is enormous. The reason is that the physiological and psychological developmental and behavioral characteristics of individuals in the core stratum are significantly different for children (elementary school level), adolescents (secondary school level), and early youth (university level), as their growth, and developmental stages continue to change. For example, the key factors that influence participation in physical activity in the target group of first-year elementary school students are extremely different across levels from those influencing the group of first-year college students. However, the existing studies tend to report school-segment similarities in terms of the content of intervention, such that the exploration of physical health promotion among students overlooks the developmental and behavioral characteristics of students at varying school levels. Therefore, the examination of physical activity behavior and intervention for the promotion of physical health among students needs to fully consider the developmental and behavioral characteristics of students across school segments.

This study aimed to analyze the current status of daily SB and PAL among students. Physical activity was categorized into low-intensity (LPA), moderate-intensity (MPA), and high-intensity (VPA) physical activities by investigating seven-day SB and PAL as well as differences in the physical activity of students aged 9–23 years. The results were analyzed according to age and school segment to provide a strong practical basis for further proposals on effective interventions.

## 2. Materials and methods

### 2.1. Participants

This study conducted tests in five schools (i.e., one elementary school, one middle school, two high schools, and one university) in Hangzhou, China, from March 2019 to December 2019. The study considered certain differences in the curriculum, academic load, and physical education class instruction across school grades. Moreover, we categorized the respondents according to grade level and analyzed trends in PALs using school grades. The survey involved students in elementary (grades 3–6), junior high (grades 7–8), high school (grades 10–11), and college (freshmen and sophomores). The test excluded students in grades 1–2 due to the effects of cognitive ability and instrument storage capacity and students in grades 9–12 due to the impact of promotion exams. Furthermore, testing excludes juniors and seniors due to the impact of graduation internships and the absence of class schedules for physical education.

A total of 467 subjects were tested using the ActiGraph accelerometer, and 83 were excluded due to invalid wear. The final sample obtained was 384 (male: 180, 46.88%). By composition, the final sample was composed of 116 elementary (grades 3–6), 79 middle (grades 7–8), 72 high (grades 10–11), and 117 college (first and second year) students. Table 1 provides detailed data on the age, weight, height, and body mass index (BMI) of the respondents. The study found significant differences between the male and female students in terms of height (*p *< 0.001), weight (*p *< 0.001), and BMI (*p *< 0.05).

**Table 1:** Participant characteristics.

	Boys			Girls			All (*n *= 384)		
Gender (female, %)	*N*	Mean	SD	*N*	Mean	SD	*N*	Mean	SD
Age	180	14.17	3.53	204	14.62	3.51	384	14.41	3.52
Height (cm)	180	162.95	18	204	156.76***	9.81	384	159.66	12.51
Weight (kg)	179	54.42	16.56	204	48.17***	11.68	383	51.09	14.49
BMI (kg/m^2^)	179	20.01	3.90	204	19.35*	3.44	383	19.66	3.67
Primary School (Grades 3–6)	58	32.2%		58	28.4%		116	30.2%	
Middle school (Grades 7–8)	39	21.7%		40	19.6%		79	20.6%	
High school (Grades 10–11)	30	16.7%		42	20.6%		72	18.8%	
University (Grades 13–14)	53	29.4%		64	31.4%		117	30.5%	
All	180	46.88%		204	53.12%		384	100%	

* *p *< 0.05.

*** *p *< 0.001.

The Medical Ethics Committee of the Department of Psychological and Behavioral Sciences in Zhejiang University approved the study [no. (2019) 001]. To be suitable for this study, the participants must be in good health, be without obvious physical defects, and complete a certain intensity of physical exercise activities. Prior to testing, all subjects were informed of the objective of the study, test items, test procedures, possible hazards, and inconveniences of the experiment. The subjects and their guardians (of elementary school students) provided written informed consent and expressed voluntary participation in this research program.

### 2.2. Measurements

The study used the ActiGraph (wGT3X-BT) model accelerometer (Pensacola Manufacturing Technologies, Pensacola, Florida, United States) to measure the daily SB and physical activity of the students. The ActiGraph accelerometer is a single-axis accelerometer with a weight of 19 g and a frequency response of 30 Hz (19), it is widely used internationally and is proved to capture the physical activity of children and adolescents (20, 21).

The instruments were numbered and paired with the subjects prior to the test, and the instruments were initialized. During this process, the height, weight, and birth date of the subjects were entered into the appropriate instruments and distributed to the subjects, who were instructed by the researchers on how to wear them on site. The subjects were instructed to wear the instrument for seven consecutive days (five school days and two rest days), except during bathing, or swimming. After the distribution of the instruments, a researcher would visit the experimental school on a daily basis as scheduled or contact the physical education teachers or parents of the students to monitor wearing.

Sampling interval (epoch): a sampling interval of 1 s was used (22). HPA in child and adolescent students typically occurs in short intervals interspersed with LPA and MPA at different times (23), using a longer sampling interval tends to underestimate the high-intensity physical activity of the students by averaging HPA and LPA. McClain (24) suggested that a sampling interval of 5 s produces the smallest root mean square error compared with longer intervals of 10, 15, 20, 30, and 60 s. Moreover, shorter sampling intervals (e.g., 1 s) should be used for the accurate measurement of the physical activity time of students across intensities (25). Therefore, the sampling interval of 1 s was selected.

Wearing area: The instrument is worn on the right hip perpendicular to the right knee joint. The measurements of physical activity were performed on children and adolescents; thus, the site of the instrument needed to be not only accurate for data acquisition but also comfortable to wear and should not interfere with the writing and learning of the wearer. In the daily measurement of physical activity, the study found no difference in the physical activity values measured when worn at the waist or at the back of the waist (26).

Cut point: The study used Evenson's algorithm (27) for the students in elementary and junior high schools to process the data and defined SB as 0–100 counts/min and LPA as 101–2,295 counts/min. The Freedeson Adult (1998) algorithm (28) was used for high school and university students to process the data, where SB, LPA, MPA, and HPA were defined as 0–100, 101–2,295, 2,296–4,011, and >4,012 counts/min, respectively. 99 counts/min, LPA was defined as 100–1,951 counts/min, MPA was defined as 1,952–5,724 counts/min, and HPA was defined as >5,725 counts/min.

Valid wear screening: The original data downloaded were first screened for validity. The existing studies stated that valid data for study should include at least two valid school test days (minimum weekdays of valid wear time: 2) and one valid weekend test day (minimum weekend days of valid wear time: 1). A valid test day should have at least 10 h of valid wear (minimum wear time per day: 600 min); a valid wear hour should be composed of >40 min of non-zero accelerometer data (29, 30). The current study screened for valid wear by including three valid school test days (minimum weekdays of valid wear time: 3), one weekend day (minimum weekend days of valid wear time: 1). School or weekend days are defined as having at least 10 h of valid wear (minimum wear time per day: 600 min) on the test day with valid wear hours composed of >40 min of non-zero accelerometer data. The valid wear time should include more than 40 min of non-zero accelerometer data.

The study assessed the PAL of the subjects using metabolic equivalents (METs), which denote the ratio of metabolic rate at work to the standard resting metabolic rate (4.184 kJ/kg/h) in which 1 MET is the resting metabolic rate at rest. Based on the total MET values of the physical activity of the subjects, physical activities with MET values ranging from 3 to 6 were categorized as MPA and those >6 were categorized and analyzed as HPA (31).

### 2.3. Statistical procedures

The study used SPSS 25.0 for Windows Statistical Package (SPSS Inc., Chicago, IL) for the statistical analysis of the test data. The Kolmogorov–Smirnov test and histograms were used to verify whether or not the data were normally distributed. Mean and standard deviation were used to describe the data if they conformed to a normal distribution (*M* ± SD); otherwise, median and interquartile spacing (25%–75%) were used. The gender effect was tested using the independent sample *t*-test. The effect values of the *t*-test were calculated with reference to the criteria of Cohen's *d* (32), and effect sizes (*d* values) were classified as strong (0.8), medium (0.5), and weak (0.2). Analysis of covariance (ANCOVA) was used to determine whether or not the differences in the mean values between the daily SB and physical activities of the students (i.e., LPA, moderate MPA, HPA, and time spent in MPA to HPA) by gender and school segment were statistically significant after adjusting for BMI. LSD *post hoc* tests were used for multiple comparisons after ANOVA; the strong, moderate, and weak effects of the effect size (*η_p_*^2^) should reach 0.15, 0.06, and 0.01, respectively, according to Cohen's criteria (32).

## 3. Results

### 3.1. Grade level differences in the current status of the daily physical activity of the students

The overall time for student participation in MVPA was 60.4 ± 23.48 min/day, which is higher for male students (63.47 ± 21.03 min/day) than those of female students (57.71 ± 25.18 min/day). The mean values of time spent in SB, LPA, MPA, and VPA for the male and female students indicated that the male students were less sedentary and had more daily activities than did the female students. A combination of factors, such as academic pressure and physical and mental development, influenced the physical activity, and SB of students across grades. Moreover, the study observed gender differences in attitudes and habits toward physical activity. Therefore, the study was conducted to analyze the trends of physical activity and SB of students across grades and genders.

**Figure 1:**
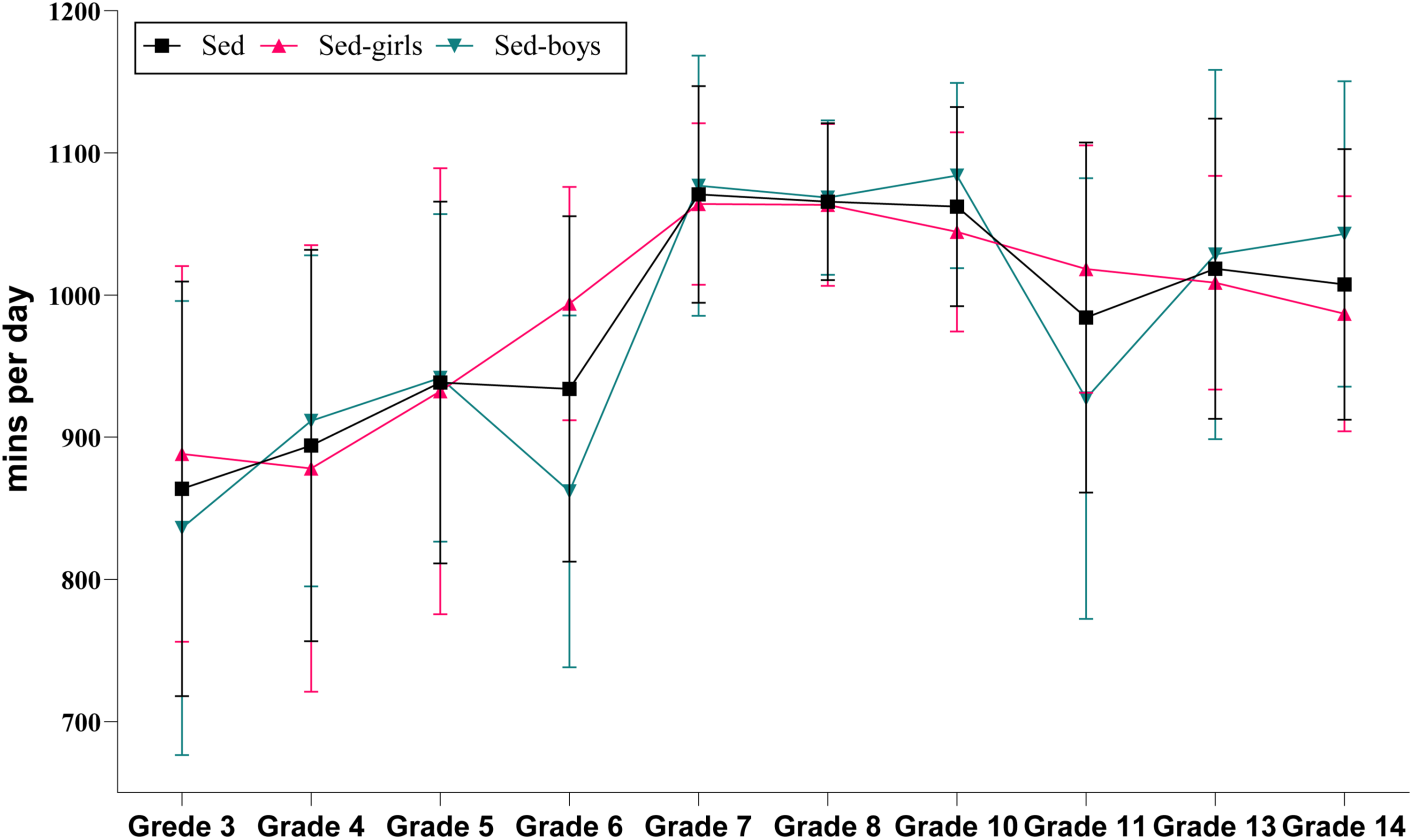
Daily sedentary behavior across grades and gender.

**Figure 2:**
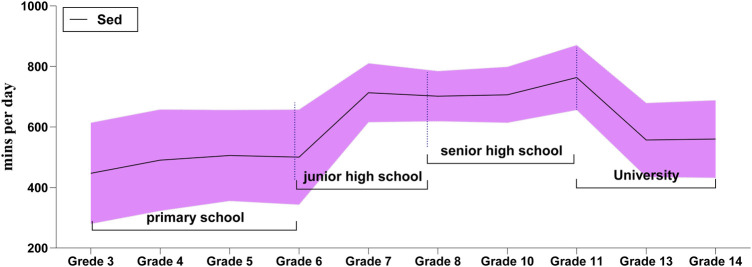
Daily sedentary behavior across grades.

Figures 1, 2 illustrate the daily changes in the length of SB among the students. The study found an increasing followed by a decreasing trend in sedentary time with the increase in age. At the elementary school level, the duration of daily SB increased with the increase in age. For example, the daily sedentary time increased by 53.09 min/day at grade 6 compared with at grade 3. The increase in daily sedentary time was significant for female students compared with that of male students in elementary school. Especially in grade 6, the daily sedentary time of the female students were 134.13 min/day higher than those of the male students. A significant increase occurred in the transition from grade 6 to middle school. In addition, the sedentary time remained higher throughout middle school. For example, the sedentary time per day was more than 3 h higher (212.85 min/day) for the first-year students than for students in grade 6. At the high school level, the curve appears at its highest point. Daily sedentary time increased from 706.17 min/day in grade 10 to 763.29 min/day in grade 11. After the transition from high school to college, the study found a significant decrease in daily sedentary time with a significant difference between male and female students in grade 14 followed by an increasing and a decreasing trend for male and female students, respectively (Table 2).

**Table 2:** Daily sedentary behavior (mins per day).

Grade	All		Girls		Boys	
	Mean	SD	Mean	SD	Mean	SD
Grade 3	447.13	167.29	512.95	127.41	373.09	179.25
Grade 4	490.39	167.76	495.56	180.50	484.84	159.58
Grade 5	505.95	150.26	567.93	154.33	472.57	142.80
Grade 6	500.22	157.28	561.19	133.88	427.06	155.75
Grade 7	713.07	97.68	712.19	76.36	713.86	115.54
Grade 8	701.68	83.12	720.44	83.72	678.23	78.65
Grade 10	706.17	92.45	681.75	87.31	736.02	92.06
Grade 11	763.29	107.41	747.67	109.30	789.33	103.40
Grade 13	556.83	122.39	559.56	103.06	554.02	140.82
Grade 14	560.37	128.49	536.41	136.06	601.45	106.58
All	598.47	162.63	584.42	180.32	610.80	144.67

**Figure 3:**
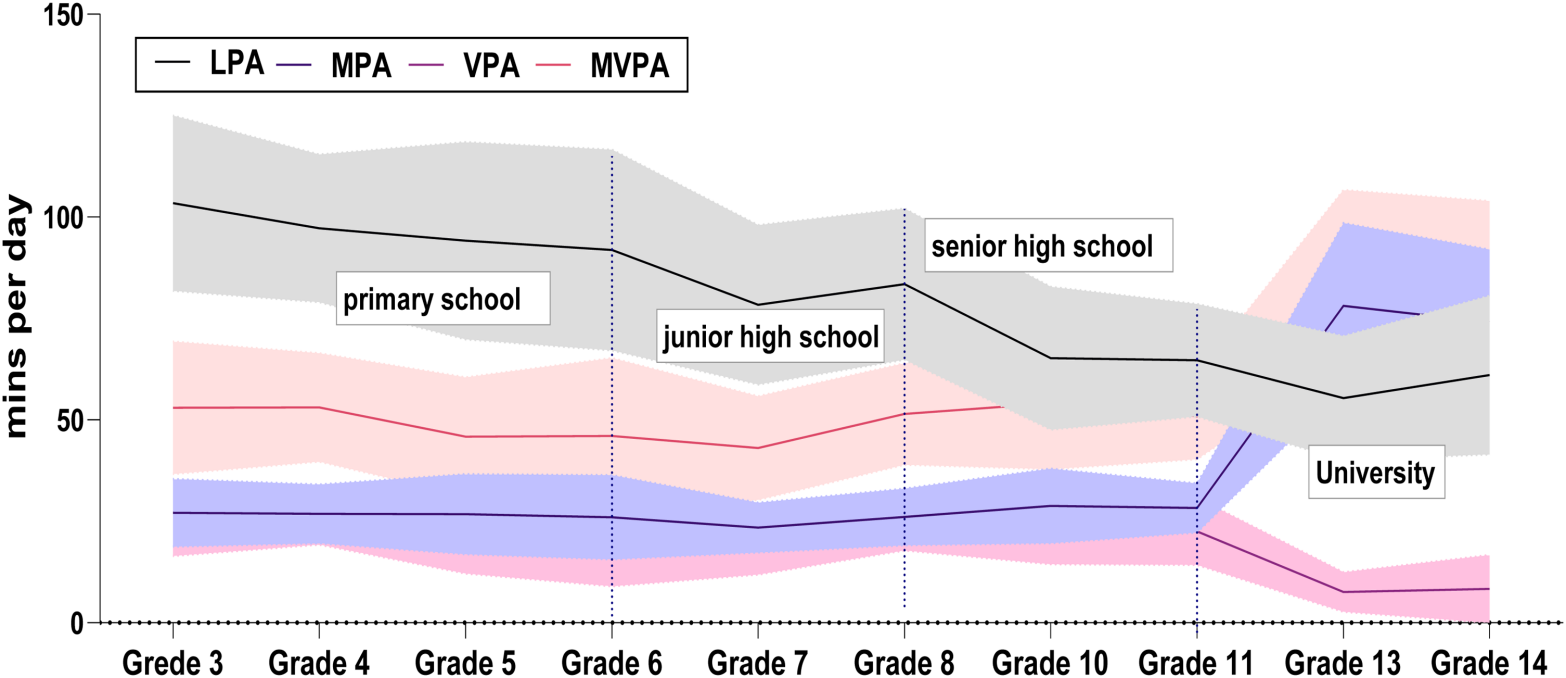
Daily physical activity across grades.

Figure 3 depicts the trend of times spent in physical activity at varying intensities per day for different grades. The results of the study indicated that the overall trend of the participation of the students in MVPA was a *W*-shape. This result is reflected in the following aspects. In elementary school, the participation of students in MVPA exhibited a decreasing trend, that is, from 50.3 min/day in grade 3 to 46.07 min/day in grade 6. In secondary school, an upward then downward fluctuation was observed. The PALs of the students continued to decline after the end of elementary school. The physical activity of students in grade 7 was the lowest for all grades at 43.5 min/day, which gradually increased as the students entered grade 8. The transition to the university level displayed a significant upward trend, especially in grade 13 in which it reached 85.67 min/day with a larger proportion of time spent by university students participating in MPA (78.1 min/day).

Furthermore, the study observed an overall decreasing trend in the amount of time that students participated in LPA with the increase in age. In other words, as the students became older, the amount of time they participated in LPA decreased. Students engaged in LPA for nearly twice as long in their third year (103.41 min/day) compared with that in their sophomore year (61.09 min/day). This decline was more pronounced for male students than that for female students. A point of concern is that the students did not engage in MVPA for ≥60 min/day per day as recommended by the WHO throughout elementary to high school.

### 3.2. School-segment differences in the current status of daily sedentary behavior and physical activity

Table 3 provides a comparison of the mean physical activity time (min/day) of the male and female students across school levels. The data demonstrate that the PAL of male students was higher than those of female students in elementary, middle, and high school; in the college level, the PAL of female students (86.49 min/day) was higher than that of the male students (85.26 min/day). In addition, sedentary time displayed the following trend: high school students > junior high school students > college students > elementary school students. The results also indicated that students in elementary (49.83 min/day), junior high (46.33 min/day), and high school (52.69 min/day) did not meet the WHO requirement of ≥60 min/day of daily MVPA.

**Table 3:** Descriptive statistics of sedentary behavior and physical activity among the students across school segments (mean and standard deviation).

PAL (min/d)	Sedentary			Light			Moderate			Vigorous			Moderate to Vigorous		
	Boys	Girls	All	Boys	Girls	All	Boys	Girls	All	Boys	Girls	All	Boys	Girls	All
Primary School (Grades 3–6)	436.3, 162.9	530.1, 146.9	483.2, 161.5	105.4, 23.1	88.6, 18.5	97.0, 22.4	30.6, 9.1	22.7, 6.6	26.7, 8.9	26.0;10.1	20.3, 7.9	23.2, 9.4	56.6, 16.8	43.1, 13.1	49.8, 16.4
Middle school (Grades 7–8)	698.9, 102.0	716.3, 79.2	707.8, 90.9	87.3, 17.7	74.5, 18.8	80.7, 19.2	26.8, 6.3	22.6, 6.3	24.7, 6.6	25.1, 9.1	19.5, 5.9	22.2, 8.1	50.6, 16.0	42.2, 10.9	46.3, 14.2
High school (Grades 10–11)	757.3, 98.6	713.1, 102.7	731.6, 102.7	65.8, 14.1	64.5, 17.3	65.0, 15.9	27.1, 6.8	29.6, 8.5	28.6, 7.9	29.0, 10.5	20.7, 7.7	24.1, 9.8	56.1, 13.0	50.3, 14.4	52.7, 14.0
University (Grades 13–14)	566.6, 133.3	550.9, 116.0	558.0, 123.9	52.4, 14.4	61.3, 17.9	57.3, 16.9	73.9, 16.6	79.2, 21.7	76.8, 19.7	9.5, 9.4	6.4, 3.9	7.8, 7.1	85.3, 20.1	86.5, 22.4	85.9, 21.3

After controlling for BMI, the results of ANCOVAs displayed a significant school-segment effect for SBs (*F *= 83, *p *< 0.001, *η_p_*^2^ = 0.4) and physical activity (LPA: *F* = 108.61, *p *< 0.001, *η_p_*^2^ = 0.47; MPA: *F* = 401.65, *p *< 0.001, *η_p_*^2^ = 0.76; VPA: *F* = 88.43, *p *< 0.001, *η_p_*^2^ = 0.42; MVPA: *F* = 118.42, *p *< 0.001, *η_p_*^2^ = 0.49). We also found a significant gender effect for LPA (*F *= 6.02, *p *< 0.05, *η_p_*^2^* *= 0.02), VPA (*F *= 45.98 *p *< 0.001, *η_p_*^2^* *= 0.11), and MVPA (*F *= 14.08, *p *< 0.05, *η_p_*^2^* *= 0.04). The study found significant interactions between school segment and gender in the mixed ANCOVA for SB (*F *= 5.61 *p *< 0.05, *η_p_*^2^* *= 0.04), LPA (*F *= 12.31 *p *< 0.001, *η_p_*^2^* *= 0.09), MPA (*F *= 6.01 *p *< 0.001, *η_p_*^2^* *= 0.05), and MVPA (*F *= 3.64 *p *< 0.05, *η_p_*^2^* *= 0.03). *Post hoc* tests revealed that the male students produced significantly higher LPA levels at 4.63 min/day (*p *= 0.015), VPA levels at 5.855 min/day (*p *< 0.001), and MVPA levels at 6.674 min/day (*p *< 0.001) than those for female students. Table 4 provides the specific results.

**Table 4:** Analysis of covariance of daily sedentary behavior and physical activity among students across school segments.

	School segment		Gender		School segment^*^ Gender	
	*F*	*η_p_* ^2^	*F*	*η_p_* ^2^	*F*	*η_p_* ^2^
Sedentary	83	0.40***	0.55	0.01	5.61	0.04*
Light	108.61	0.47***	6.02	0.02*	12.31	0.09***
Moderate	401.65	0.76***	0.53	0.00	6.01	0.05***
Vigorous	88.43	0.42***	45.98	0.11***	1.26	0.01
Moderate–Vigorous	118.22	0.49***	14.08	0.04***	3.64	0.03*

*p*-Value is the result of the covariance test, where BMI is a covariate and school-segment and gender are fixed factors.

* *p* < 0.05.

*** *p* < 0.001.

**Figure 4:**
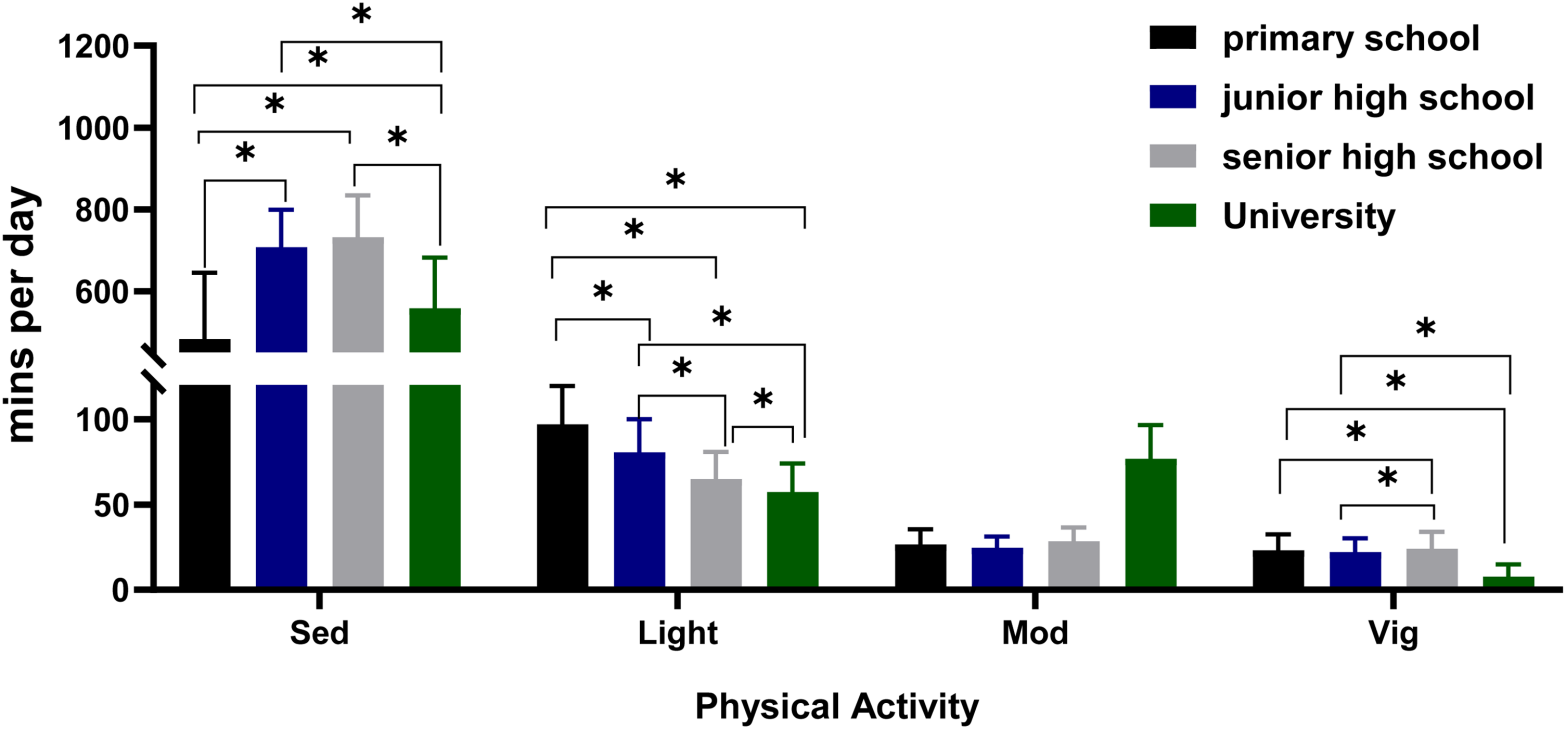
Daily sedentary behavior and physical activity across school segments.

Variables with significant differences were compared using the Bonferroni's *post hoc* test to further understand the differences across the four school segments (Figure 4). The study found that the sedentary time of middle school students was greater than that of elementary school students at 234.363 min/day (95% CI* *= 197.25–271.48, *p *< 0.001) and college students at 143.51 min/day (95% CI* *= 107.20–179.82, *p *< 0.001). Primary school students displayed higher levels of LPA than those of middle school students at 19.046 min/day (95% CI* *= 13.75–24.34, *p *< 0.001). High school students at 37.048 min/day (95% CI* *= 31.24–42.86, *p *< 0.001), and college students at 44.563 min/day (95% CI* *= 39.52–49.61, *p *< 0.001). University students produced higher levels of MPA than those of elementary school students at 48.967 min/day (95% CI* *= 45.43–52.50, *p *< 0.001), middle school students at 51.625 min/day (95% CI* *= 48.0–55.26, *p *< 0.001), and high school students at 48.56 (95% CI* *= 44.83–52.28, *p *< 0.001). In addition, the study found that VPA was greater for high school students than those for elementary school students at 2.714 min/day (95% CI* *= 0.06–5.37, *p *< 0.05), middle school students at 2.956 min/day (95% CI* *= 0.28–5.63, *p *< 0.005), and college students at 16.975 min/day (95% CI = 14.54–19.41, *p *< 0.001).

For MVPA, the study found an interaction between gender and school segment. Therefore, the study separated the male and female students for a two-by-two comparison per school segment. The MVPA of female students in college was greater than that of female students in elementary school at 41.141 min/day (95% CI = 34.67–47.61, *p *< 0.001), middle school at 43.245 min/day (95% CI = 36.68–49.81, *p *< 0.001), and high school at 35.893 min/day (95% CI = 29.33–42.45, *p *< 0.001). The MVPA of male students in college was greater than those of male students in elementary school at 28.199 min/day (95% CI = 21.35–35.04, *p *< 0.001), middle school at 32.399 min/day (95% CI = 25.26–39.54, *p *< 0.001), and high school at 27.857 min/day (95% CI = 20.35–35.36, *p *< 0.001).

## 4. Discussion

The present study found that the overall mean MVPA (60.40 min/day) of university, secondary, and elementary school students met the recommended amount of physical activity (MVPA ≥ 60 min/day) proposed by the WHO. In the total sample, 41.2% of the children and adolescents met this recommended amount. The current findings were higher than those of previous studies, such as Wang, who found that only 5.6% met the recommended amount after testing 3,121 students from second grade to second grade of high school in 11 cities across China (7). The reason for this result may be due to the large geographical differences in the PALs of the students. Yu (33) observed a geographical variability in the PALs of students, which is influenced by local economic, cultural, educational, and physical education class settings after a survey of students in the Zhejiang, Guangdong, Beijing, and Yunnan provinces of China.

The current survey demonstrated that the total sample spent 598.47 ± 162.63 min/day (approximately 10 h) in daily SB. Studies in other countries illustrated that adolescents spend approximately 6–8 h per day in a sedentary position (34–36), and the findings of the present study are greater than those of studies conducted in other countries. This result pointed to excessive daily SB among the students. Physical inactivity not only refers to the lack of health-promoting physical activities, such as housework, and commuting, but also includes another important concept, that is, SB. This behavior cannot be simply understood as the lack of health-promoting physical activities (i.e., moderate and above-intensity physical activity); instead, it is a state of behavior characterized by continued and prolonged sitting and lying down and the lack of whole-body activities such as sitting still and watching television (37). SB is generally considered to be no more than 1.5 METs in intensity (38). Tremblay et al. (39) conducted a systematic review of 232 studies and concluded that a dose–effect relationship exists between SB and health risks in school-age children and adolescents (ages 5–17 years), where an SB of >2 h/day leads to changes in body composition, decreased body mass, low levels of self-esteem, and pro-social behavior. Evenson et al. (40) defined a sustained sedentary session as ≥30 min in which approximately 80% of the time is spent on less than 100 steps per min.

The study found a significant variability in physical activity across school levels. Measured using the ActiGraph accelerometer, the results demonstrated the following trend: college male students > elementary school male students > junior high school male students > high school and college female students > high school female students > elementary school female students > junior high school female students. Specifically, the study observed the following trends for VPA: high school students > elementary school students > junior high school students > college students and MVPA: college students > high school students > elementary school students > junior high school students. Troiano et al. (41) analyzed accelerometer data from a representative nationwide health and nutrition survey conducted in the United States and found that PALs tended to progressively decline from the childhood to the adolescent stages, with attainment decreasing from 42% at ages 6–11 years to 8% at the adolescent stage and continuing to decline into adulthood. A study with a sample size of 1,032 children (aged 9 years at baseline testing) measured by an accelerometer and followed up for 6 years found that MVPA decreased from 180 min/day at age 9 years to 50 min/day at age 15 years with a decreasing trend of 35 min/day per year (42). A national survey in Canada on 21,271 children (aged 5–12 years) and 12,956 adolescents (aged 13–19 years) used pedometer measurements and found that step counts peaked at age 10 years and gradually decreased thereafter (43). Evidently, the gradual decrease in physical activity as children transition from childhood to adolescence (41, 42) is a widespread international problem that warrants scholarly attention.

During the survey, the study found that one of the high schools, with the unique advantage of being built on a mountain, arranges a weekly 40 min hillside run for the class as a group. The school offered 100% attendance for extra credit in physical education class and 80% attendance for passing. If the students did not achieve the 90% attendance, they would be scheduled to compensate for the run at the school playground at the end of the period. Moreover, a time limit was observed for the entire phase of the hill run in which each class has its own timeline and needs to return at the specified time. During the survey, after interviewing the students who had just finished running, the researchers learned that the hill running activities were extremely intense and that the students felt exhausted after each run, which explains the reason for why these high school students produced the most participation time in HPA.

In addition, one of the universities developed a program called “Campus Sunshine Run Intelligent Management System” with the objective of improving the physical health of its students, forming exercise behaviors, and developing a healthy lifestyle. Through this system, the students can participate in *independent* and *self-help* activities in the management of sunshine long-distance running and implement *scientific* and *in-class and out-of-class* teaching of sunshine long-distance running. The system adopted a full wireless network mode, through which the students can participate the independent and self-help management. Moreover, the system adopted the full wireless network mode to realize wireless data collection, which covers the entire campus through a self-organized network. Swipe card terminals distributed in the whole school wirelessly transmitted the sports swipe card information of the students to the base station for receiving data. It also used face color monitoring technology to achieve an anti-cheating management function in monitoring the students. The university required that freshman and sophomore students conduct sunshine long-distance running exercise for 10 weeks per semester. Each long run is no more than 1,500 m for female students and 2,000 m for boys. The students can obtain 100 points for a total of 30 runs per semester or 20 runs to pass (60 points), which is more than the required number of times for giving additional points. The long-distance running score accounted for 20% of the total physical education score. During the survey, the students were interviewed after the physical education class and learned that the majority of them could maintain the frequency of participating in long-distance running at twice per week, which well explains why the college students displayed the longest duration of time for MPA.

This is a study that investigated the physical activity of school-age children from 9 to 23 years old using accelerometer measurements and analyzed the characteristics of the variability of physical activity behaviors of students in different age groups. In terms of measurement methods this study circumvents the disadvantages of survey methods such as physical activity questionnaires and physical activity diary recall records that are subjectively influenced and difficult to recall. However, it also has some limitations. Due to the vast territory of China and the difficulty of sampling, the survey population did not cover all regions of China. In order to make the survey population more representative, students from urban schools, suburban schools, and schools in combined suburban areas were selected for this study.

## 5. Conclusions

This cross-sectional study used the accelerometer for tracking the daily SB and physical activity of the students for one week and found that they were physically inactive and mainly sedentary. Moreover, the study found the following trend, that is, participation in MVPA time decreased, and sedentary time increased with the increase in age. It further a variability in the SB and physical activity of the students in age and school segment. Based on the behavioral characteristics of students across school segments, this study concluded that interventions targeting students' physical activity and physical health should be school segment specific. The results of the study provide a basis for future analysis of factors influencing students' physical activity behaviors across school segments and for proposing targeted intervention strategies for the future.

## Data Availability

The raw data supporting the conclusions of this article will be made available by the authors, without undue reservation.
